# Coinfections with Other Respiratory Pathogens among Patients with COVID-19

**DOI:** 10.1128/spectrum.00163-21

**Published:** 2021-07-21

**Authors:** K. Sreenath, Priyam Batra, E. V. Vinayaraj, Ridhima Bhatia, KVP SaiKiran, Vishwajeet Singh, Sheetal Singh, Nishant Verma, Urvashi B. Singh, Anant Mohan, Sushma Bhatnagar, Anjan Trikha, Randeep Guleria, Rama Chaudhry

**Affiliations:** a Department of Microbiology, All India Institute of Medical Sciences, New Delhi, India; b Department of Anaesthesiology, Pain Medicine and Critical Care, All India Institute of Medical Sciences, New Delhi, India; c Department of Geriatric Medicine, All India Institute of Medical Sciences, New Delhi, India; d Department of Hospital Administration, National Cancer Institute, Jhajjar, Haryana, India; e Department of Pulmonary, Critical Care and Sleep Medicine, All India Institute of Medical Sciences, New Delhi, India; f Department of Onco-Anaesthesia and Palliative Medicine, All India Institute of Medical Sciences, New Delhi, India; Brigham and Women's Hospital, and Harvard Medical School

**Keywords:** COVID-19, SARS-CoV-2, coinfections, multiple-pathogen testing, respiratory PCR

## Abstract

Emerging evidence indicates that severe acute respiratory syndrome coronavirus 2 (SARS-CoV-2)-infected individuals are at an increased risk for coinfections; therefore, physicians need to be cognizant about excluding other treatable respiratory pathogens. Here, we report coinfection with SARS-CoV-2 and other respiratory pathogens in patients admitted to the coronavirus disease (COVID) care facilities of an Indian tertiary care hospital. From June 2020 through January 2021, we tested 191 patients with SARS-CoV-2 for 33 other respiratory pathogens using an fast track diagnostics respiratory pathogen 33 (FTD-33) assay. Additionally, information regarding other relevant respiratory pathogens was collected by reviewing their laboratory data. Overall, 13 pathogens were identified among patients infected with SARS-CoV-2, and 46.6% (89/191) of patients had coinfection with one or more additional pathogens. Bacterial coinfections (41.4% [79/191]) were frequent, with Staphylococcus aureus being the most common, followed by Klebsiella pneumoniae. Coinfections with SARS-CoV-2 and Pneumocystis jirovecii or Legionella pneumophila were also identified. The viral coinfection rate was 7.3%, with human adenovirus and human rhinovirus being the most common. Five patients in our cohort had positive cultures for Acinetobacter baumannii and K. pneumoniae, and two patients had active Mycobacterium tuberculosis infection. In total, 47.1% (90/191) of patients with coinfections were identified. The higher proportion of patients with coinfections in our cohort supports the systemic use of antibiotics in patients with severe SARS-CoV-2 pneumonia with rapid de-escalation based on respiratory PCR/culture results. The timely and simultaneous identification of coinfections can contribute to improved health of COVID-19 patients and enhanced antibiotic stewardship during the pandemic.

**IMPORTANCE** Coinfections in COVID-19 patients may worsen disease outcomes and need further investigation. We found that a higher proportion of patients with COVID-19 were coinfected with one or more additional pathogens. A better understanding of the prevalence of coinfection with other respiratory pathogens in COVID-19 patients and the profile of pathogens can contribute to effective patient management and antibiotic stewardship during the current pandemic.

## INTRODUCTION

A cluster of mysterious viral pneumonia cases, later named coronavirus disease 2019 (COVID-19), was first identified in Wuhan city, China, in December 2019 (1). The virus spread rapidly beyond Wuhan city and has affected 223 countries, areas, or territories and infected more than 137 million people globally (14 April 2021) (https://covid19.who.int/) (2). India has become the second worst-affected country by COVID-19, with 14.1 million reported cases and 173,000 deaths as of 14 April 2021 (https://www.mohfw.gov.in/). The clinical spectrum of COVID-19 varies from asymptomatic infection to severe pneumonia and systemic manifestations, including sepsis, septic shock, and multiorgan dysfunction syndrome (3). Bacterial and viral coinfections are frequently reported in COVID-19 patients and can lead to increased morbidity and mortality rates (4). Among COVID-19 patients, the prevalence of coinfections may vary, and the proportions could be up to 50% among nonsurvivors (5).

When both innate and adaptive immunity become impaired due to a previous viral infection, including COVID-19, bacteria can utilize this temporarily compromised host immune condition and cause secondary pneumonia (6). In a meta-analysis, Lansbury and colleagues reported bacterial coinfection in 7% of patients with COVID-19, and higher coinfection rates were observed in intensive care unit (ICU) patients than in those in hospital wards (7). The commonly reported bacterial pathogens in patients with severe acute respiratory syndrome coronavirus 2 (SARS-CoV-2) infection include Staphylococcus aureus, Streptococcus pneumoniae, Klebsiella pneumoniae, Legionella pneumophila, Mycoplasma pneumoniae, and Chlamydophila pneumoniae (8). A few studies have reported rates of coinfection with other respiratory viruses ranging from 0 to 20% in patients with COVID-19 (9). Common coinfecting viruses include influenza A virus (IAV), coronavirus, rhinovirus/enterovirus (EV), metapneumovirus, parainfluenza virus, influenza B virus (IBV), and respiratory syncytial virus (RSV) (10). Even though few studies have captured the data on bacterial and viral coinfections, more information in this regard is urgently required, especially from the Indian subcontinent.

In Chinese cohorts, ∼60 to 70% of patients hospitalized with COVID-19 received empirical broad-spectrum antibiotics due to suspected or confirmed bacterial coinfections (11, 12). However, the overuse of antibiotics may cause adverse effects associated with bacterial drug resistance. Therefore, the adequate use of antibiotics and antibiotic stewardship (ABS) approaches is warranted during the ongoing COVID-19 pandemic (13). ABS policies must focus on prescribing an optimal empirical antibiotic and rapid de-escalation based on microbiological reports (14). The identification of bacterial coinfections in COVID-19 patients can provide pathogen-targeted therapy and minimize the negative consequences of antibiotic overuse.

In the present study, we prospectively analyzed 191 patients with COVID-19 admitted to the COVID care facilities of an Indian tertiary care hospital with the specific aim to determine bacterial and viral coinfections, simultaneously.

## RESULTS

### Patient characteristics.

A total of 191 laboratory-confirmed COVID-19 patients were evaluated for other respiratory pathogens using the fast track diagnostics respiratory pathogen 33 (FTD-33) assay. The baseline demographics, clinical characteristics, patient comorbidities, complications during the hospital course, and clinical outcomes of all case-patients enrolled in the present study are shown in Table 1. Twelve (6.2%) out of 191 patients were asymptomatic cases, 57 (29.8%) had mild infections, 39 (20.4%) had moderate infections, and 83 (43.5%) had severe infections, according to Indian Council of Medical Research (ICMR) criteria (clinical management protocol COVID-19, version 5) (15). Overall, 135 (70.6%) patients had pneumonia, including 96 (50.2%) patients with acute respiratory distress syndrome (ARDS) and 52 (27.22%) who developed septic shock. In total, there were 69 (36.1%) deaths in the cohort. The median length of hospital stay was 13 days (range, 1 to 46 days). The details of other microbiological investigations performed in a few patients upon hospitalization are shown in Table 1.

**TABLE 1:** Baseline demographics and clinical characteristics of COVID-19 patients enrolled (*n* = 191)^*a*^

Characteristic	Value
Demographics	
No. (%) of male patients	137 (71.7)
Median age (yrs) (IQR)	50 (15–86)

No. (%) of patients with major comorbidity(ies)	
At least one comorbid condition	140 (89)
Hypertension	64 (33.5)
Diabetes mellitus	61 (31.9)
Renal disease	35 (18.3)
Cardiovascular diseases	29 (15.2)
Malignancy	16 (8.3)
Chronic respiratory diseases	13 (6.8)

No. (%) of patients with symptom at presentation	
Fever	169 (88.4)
Cough	115 (60.2)
Shortness of breath	112 (58.6)
Confusion	36 (18.8)
Abdominal pain/diarrhea	19 (9.9)
Headache	14 (7.3)

No. of patients with positive chest radiography findings (%)	96/111 (86.4)

Laboratory findings	
No. (%) of patients with abnormal hemoglobin^*b*^	148 (77.5)
Median hemoglobin level (g/dl) (IQR)	10.8 (4.0–16)
No. (%) of patients with leukocytosis^*c*^	91 (41.6)
No. (%) of patients with lymphopenia^*d*^	137 (71.7)
No. (%) of patients with thrombocytopenia^*e*^	72 (37.6)
No. (%) of patients with elevated AST^*f*^	110 (57.5)
No. (%) of patients with elevated ALT^*g*^	80 (41.9)
No. (%) of patients with C-reactive protein level of >6 mg/dl	80/166 (48.2)
Median C-reactive protein level (mg/dl) (IQR)	5 (0.025–37)
No. (%) of patients with abnormal interleukin-6^*h*^	123/148 (83.1)
No. (%) of patients with procalcitonin level of >0.1 ng/ml	76/109 (69.7)
Median procalcitonin level (ng/ml) (IQR)	0.41 (0.01–100)
No. (%) of patients with elevated D-dimer^*i*^	5/99 (5)
No. (%) of patients with abnormal blood urea nitrogen^*j*^	87 (45.5)
No. (%) of patients with abnormal creatinine^*k*^	90 (47.1)
No. (%) of patients with abnormal serum ferritin^*l*^	101/160 (63.1)
No. (%) of patients with microbiological diagnosis	
Blood cultures collected	24 (12.6)
Blood cultures positive	0
No. (%) of FTD respiratory pathogen 33-positive patients	89 (46.6)
Respiratory samples collected for culture	13 (6.8)
Clinically relevant pathogen in respiratory samples by culture	5 (38.5)
No. of bacterial pathogens	
Acinetobacter baumannii	4
Klebsiella pneumoniae	1
No. (%) of multiplex PCR (FTD-33 assay)- or culture-positive patients	90 (47.1)
No. (%) of patients with coinfection with respect to duration of specimen collected for testing	
Sample collected after >48 h of hospital admission	68/146 (46)
Sample collected in the 1st 48 h of hospital admission	22/45 (48.9)
Legionella pneumophila urinary antigen test performed	191 (100)
Legionella pneumophila urinary antigen test positive	0
No. (%) of patients with SARS-CoV-2 identification	
Positive by real-time RT-PCR	101 (52.8)
Positive by CB-NAAT	70 (36.6)
Positive by rapid antigen detection	20 (10.4)

No. (%) of patients with treatment	
Empirical antibiotic	152/177 (85.9)
Penicillins and cephalosporins	31 (17.5)
Tetracyclines	52 (29.3)
Macrolides	19 (9.9)
Glycopeptides	50 (26.2)
Carbapenems	40 (22.6)
Combination antibiotics	80 (41.88)
Fluoroquinolones	10 (5.6)
Antiviral therapy	28 (15.8)

Clinical outcomes	
Median length of hospitalization (days) (IQR)	13 (1–46)
No. (%) of patients who required ICU admission	139 (72.77)
No. (%) of patients who required ventilatory support	99 (51.8)
Mortality rate [no. (%) of patients]	69 (36.1)

a ALT, alanine aminotransferase; AST, aspartate aminotransferase; CB-NAAT, cartridge-based nucleic acid amplification test; ICU, intensive care unit; RT-PCR, reverse transcription-PCR.

b Reference values are 12 to 15 g/dl for men and 13 to 17 g/dl for women.

c The reference range is 4 × 10^3^ to 11 × 10^3^ cells/μl.

d The reference range is 20 to 40%.

e The reference range is 150 × 10^3^ to 400 × 10^3^ cells/μl.

f The reference range is 5 to 40 U/liter.

g The reference range is 5 to 42 U/liter.

h The reference range is 5 to 15 pg/ml.

i The reference value is <500 ng/μl.

j The reference range is 10 to 50 mg/dl.

k The reference range is 0.5 to 1.2 mg/dl.

l The reference range 10 to 291 ng/ml.

Treatment information was available for only 177 (92.6%) patients in our cohort (Table 1). Most patients (152/177 [85.8%]) were administered antibiotics, commonly combination antibiotics with broad-spectrum coverage (either the amoxicillin/clavulanate combination, piperacillin/tazobactam combination, or cefoperazone/sulbactam combination [*n* = 80 {45.2%}]). Forty-two (23.7%) patients received corticosteroids, mainly methylprednisolone therapy.

### Coinfection with respiratory pathogens in SARS-CoV-2. (i) Multiplex real-time RT-PCR (FTD-33 assay).

Among the 191 patients tested, 89 (46.5%) had viral, bacterial, or fungal coinfection identified by the FTD-33 assay (Table 2). In total, 14 (7.3%) patients had other respiratory viral coinfections (viral only, viral-bacterial, or viral-fungal), and 79 (41.4%) had bacterial coinfections (bacterial only, bacterial-viral, or bacterial-fungal). Five (2.6%) patients had coinfection with Pneumocystis jirovecii (Table 2). Specifically, a single virus (other than SARS-CoV-2) was detected in 8 samples, and a single bacterium was identified in 62 samples in patients with SARS-CoV-2. Multiple codetections were present in 19 samples, including 13 double detections (mixed bacterial, *n* = 7; bacterial-viral, *n* = 4; bacterial-fungal, *n* = 2) and 6 triple detections (mixed bacterial, *n* = 3; bacterial-fungal, *n* = 2; bacterial-viral, *n* = 1).

**TABLE 2:** Single and multiple coinfections in patients with SARS-CoV-2 identified by the FTD-33 assay (*n* = 191)^*a*^

Characteristic of infection	No (%) of patients				
	Total (*n* = 191)	Asymptomatic (*n* = 12 [6.3%])	Mild (*n* = 57 [29.8%])	Moderate (*n* = 39 [20.4%])	Severe (*n* = 83 [43.5%])
Any pathogen	89 (46.6)	3 (25)	25 (43.9)	19 (48.7)	42 (50.6)
Any bacterium^*b*^	79 (41.3)	3 (25)	21 (36.8)	17 (43.5)	38 (45.7)
Any virus^*c*^	14 (7.3)		5 (8.8)	3 (7.6)	6 (7.2)
Any fungus^*d*^	5 (2.6)		2 (3.5)		3 (3.6)
Bacterium-virus	5 (2.6)		2 (3.5)	1 (2.5)	2 (2.4)
Bacterium-fungus	2 (1)				2 (2.4)
Virus-fungus	1 (0.52)				1 (1.2)
Bacterium-virus-fungus					

a Virus refers to any respiratory virus other than SARS-CoV-2. Fungus refers to Pneumocystis jirovecii.

b Containing bacteria only and bacterium-virus, bacterium-fungus, or bacterium-virus-fungus.

c Containing virus only and virus-bacterium, virus-fungus, or bacterium-virus-fungus.

d Containing fungus only and fungus-bacterium, fungus-virus, or fungus-virus-bacterium.

**FIG 1:**
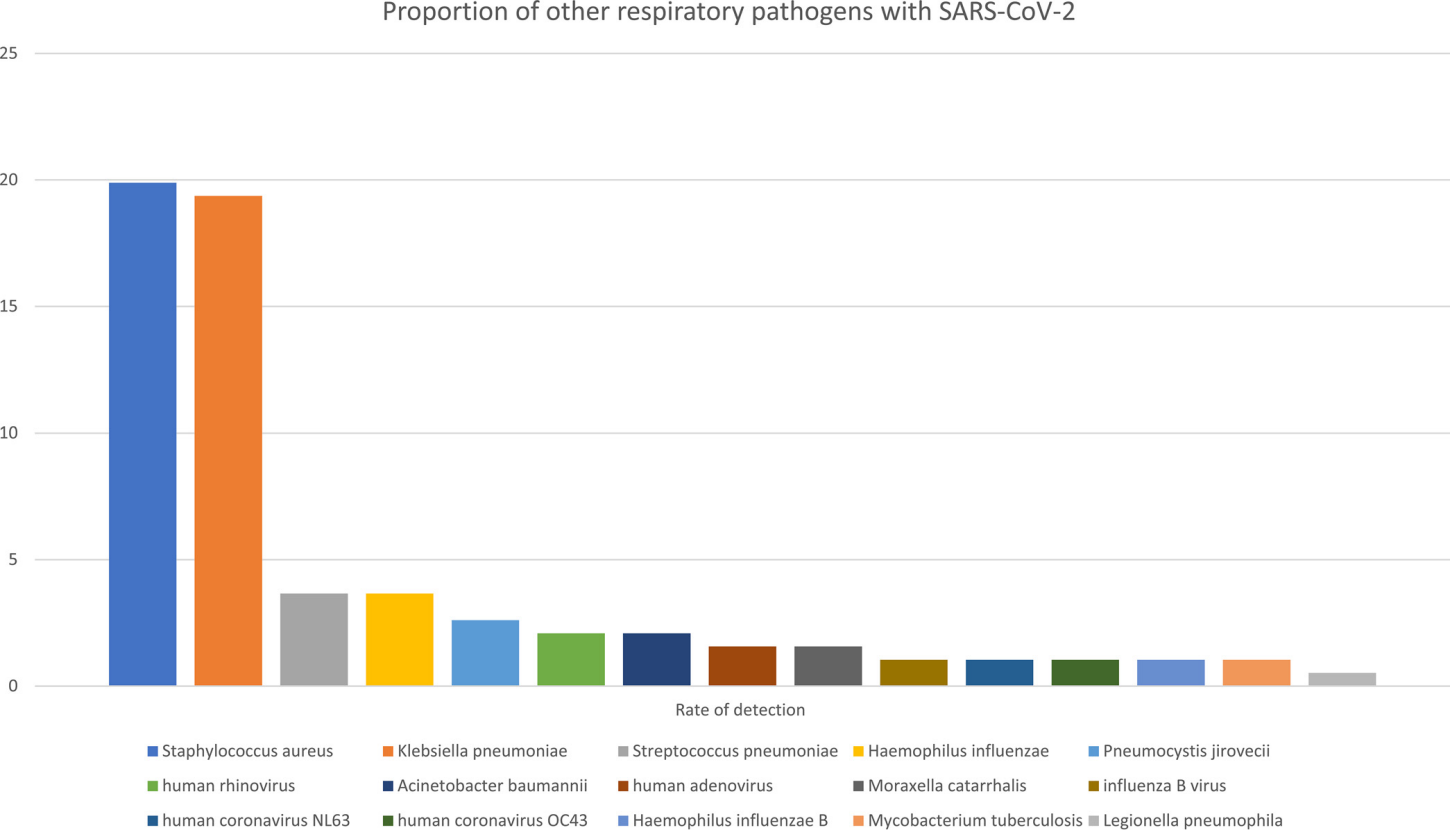
Proportions of other respiratory pathogens with SARS-CoV-2 coinfections determined by the FTD-33 assay or culture.

A total of 13 pathogens were identified, including 7 bacteria, 5 viruses, and 1 fungus (P. jirovecii) (Table 3). The coinfecting pathogens identified in this study were as follows: S. aureus (*n* = 38; 19.9%), K. pneumoniae (*n* = 37; 19.4%), S. pneumoniae (*n* = 7; 3.7%), Haemophilus influenzae (*n* = 7; 3.7%), *P. jirovecii* (*n* = 5; 2.6%), human rhinovirus (HRV) (*n* = 4; 2.1%), human adenovirus (HAdV) (*n* = 3; 1.6%), Moraxella catarrhalis (*n* = 3; 1.6%), influenza B virus (IBV) (*n* = 2; 1.1%), human coronavirus (HCoV) NL63 (*n* = 2; 1.1%), HCoV OC43 (*n* = 2; 1.1%), H. influenzae type b (Hib) (*n* = 2; 1.1%), and L. pneumophila (*n* = 1; 0.5%) (Fig. 1).

**TABLE 3:** Respiratory pathogens identified by the FTD-33 assay among the patients tested (*n* = 191)

Pathogen identified	No. of patients			
	Cases with single detection	Cases with double detection	Cases with triple detection	Total
Human rhinovirus	2	2		4
Influenza B virus		1	1	2
Human coronavirus NL63	1	1		2
Human coronavirus OC43	1	1		2
Human adenovirus	3			3
Staphylococcus aureus	25	8	5	38
Haemophilus influenzae type b	1	1		2
Streptococcus pneumoniae	3	2	2	7
Pneumocystis jirovecii	1	2	2	5
Legionella pneumophila			1	1
Klebsiella pneumoniae	27	5	5	37
Moraxella catarrhalis	2	1		3
Haemophilus influenzae	3	2	2	7

### (ii) Coinfections with patient age and disease severity.

Of the 13 pathogens identified in this study, 11 were detected in patients 15 to 44 years of age, except for HCoV NL63 and HCoV OC43. Similarly, for patients between 45 and 64 years of age and ≥65 years of age, totals of 8 and 9 pathogens were discovered, respectively. Only 5/13 pathogens, including K. pneumoniae, S. pneumoniae, S. aureus, H. influenzae, and HAdV, appeared in all these age groups. Detection rates for coinfections in asymptomatic, mild, moderate, and severe disease categories are detailed in Table 2. Overall, 4, 10, 8, and 11 pathogens were identified in the asymptomatic, mild, moderate, and severe disease cases. Only S. aureus, K. pneumoniae, and S. pneumoniae were found in all four disease categories. Detection rates of K. pneumoniae, S. aureus, S. pneumoniae, *P. jirovecii*, L. pneumophila, HCoV NL-63, and HCoV OC-43 were higher in the severe disease category, although this difference was not statistically significant.

### (iii) Identification of coinfections by other methods.

By retrospectively reviewing the medical records, it was found that respiratory cultures were performed for 13/191 (6.8%) patients. Bacterial pathogens were isolated in only 5/13 (38.5%) patients (Table 1). Four patients had positive respiratory cultures for Acinetobacter baumannii. The antimicrobial susceptibility testing results for these isolates showed that the three strains of A. baumannii were resistant to all the tested antibiotics except colistin. The remaining strain was susceptible only to colistin and the cefoperazone/sulbactam combination. One patient had a positive culture for K. pneumoniae, and the isolate was susceptible to all the antibiotics tested. Two patients in our cohort tested positive for M. tuberculosis by Xpert MTB/RIF, a cartridge-based nucleic acid amplification test (CB-NAAT) for the simultaneous detection of M. tuberculosis and resistance to rifampicin. Of the two M. tuberculosis isolates detected, one was resistant to rifampicin. Of the 7 patients for whom an additional respiratory pathogen (A. baumannii, K. pneumoniae, and M. tuberculosis) was identified, 6 were positive by the FTD assay for other bacteria (S. aureus and K. pneumoniae). Only one patient with a positive culture for A. baumannii tested negative by the FTD assay (target not included in the panel). Therefore, in total, 90/191 (47.1%) patients had coinfections identified by multiplex respiratory PCR or culture. Altogether, multiple codetections were present in 24 patients, including 18 double detections (mixed bacterial, *n* = 12; bacterial-viral, *n* = 4; bacterial-fungal, *n* = 2) and 6 triple detections (mixed bacterial, *n* = 3; bacterial-fungal, *n* = 2; bacterial-viral, *n* = 1). Pathogen combinations identified among all multiple codetections (*n* = 24) are shown in Table 4.

**TABLE 4:** Pathogen combinations identified among patients with SARS-CoV-2 by the FTD-33 assay or respiratory culture^*a*^

Pathogen	No. of patients coinfected by:													
	HRV	IBV	HCoV NL-63	HCoV OC43	S. aureus	H. influenzae type b	S. pneumoniae	P. jirovecii	L. pneumophila	K. pneumoniae	M. catarrhalis	H. influenzae	A. baumannii	M. tuberculosis
Human rhinovirus (*n* = 4)	NA				1		1							
Influenza B virus (*n* = 2)		NA			1	1				1				
Human coronavirus NL63 (*n* = 2)			NA							1				
Human coronavirus OC43 (*n* = 2)				NA				1						
S. aureus (*n* = 25)	1	1			NA		2	3	1	7	1	2		1
H. influenzae type b (*n* = 2)		1				NA								
S. pneumoniae (*n* = 7)	1				2		NA			2		1		
P. jirovecii (*n* = 5)				1	3			NA	1	1				
L. pneumophila (*n* = 1)					1			1	NA					
K. pneumoniae (*n* = 37)		1	1		7		2	1		NA		3	3	1
M. catarrhalis (*n* = 3)					1						NA			
H. influenzae (*n* = 7)					2		1			3		NA		
A. baumannii (*n *= 4)										3			NA	
M. tuberculosis (*n *= 2)					1					1				NA

a Including 18 patients with double detections and 6 patients with triple detections by the FTD-33 assay or respiratory culture. HRV, human rhinovirus; IBV, influenza B virus; HCoV, human coronavirus; NA, not applicable.

### Characteristics of patients coinfected with SARS-CoV-2.

Table 5 shows a comparison of patients with SARS-CoV-2 with and without coinfections. None of the demographic and clinical characteristics showed a significant difference between patients with and those without coinfections. Of the laboratory findings, significantly more patients with coinfections had abnormal serum creatinine levels (*P *= 0.001), lower platelet counts (*P* = 0.044), and higher C-reactive protein (CRP) levels (*P* = 0.025) than those infected with only SARS-CoV-2 (Table 5).

**TABLE 5:** Characteristics of patients with COVID-19 with and without coinfections in a tertiary care hospital in India from 2020 to 2021^*a*^

Characteristic	Value for group		*P* value
	Patients with no coinfections (*n* = 101)	Patients with coinfections (*n* = 90)	
Median age (yrs) (range)	50 (15–85)	50 (17–86)	0.846
No. (%) of male patients	72 (67.9)	65 (72.2)	0.886
No. (%) of patients with concurrent condition	70 (69.3)	70 (77.8)	0.187
Hypertension	29 (28.7)	35 (38.9)	0.137
Diabetes mellitus	27 (26.7)	34 (37.7)	0.102
Renal disease	14 (13.8)	21 (23.3)	0.091
Cardiovascular disease	15 (14.8)	14 (15.5)	0.892
No. (%) of patients with sign(s) or symptom			
Fever	88 (87.1)	81 (90)	0.535
Cough	62 (61.3)	53 (58.8)	0.725
Dyspnea	57 (56.4)	55 (61.1)	0.513
Confusion	17 (16.8)	19 (21.1)	0.45
Headache	7 (6.9)	7 (7.7)	0.823
Myalgia	10 (9.9)	13 (14.4)	0.336
Abdominal pain or diarrhea	9 (8.9)	10 (11.1)	0.612
Positive chest radiography findings	51/60 (85)	45/51 (88.24)	0.619
Bilateral infiltrations	37/60 (61.7)	28/51 (54.9)	0.471
Pulmonary consolidations	7/60 (11.7)	9/51 (17.6)	0.371
Pleural effusions		3/51 (5.9)	0.094
Laboratory parameters			
No. (%) of patients with abnormal hemoglobin^*b*^	75 (74.2)	73 (81.1)	0.198
No. (%) of patients with leukocytosis^*c*^	48 (47.5)	43 (47.8)	0.913
No. (%) of patients with lymphopenia^*d*^	77 (76.2)	60 (66.7)	0.321
No. (%) of patients with thrombocytopenia^*e*^	32 (31.7)	40 (44.4)	0.060
No. (%) of patients with elevated AST^*f*^	56 (55.4)	54 (60)	0.358
No. (%) of patients with elevated ALT^*g*^	41 (40.6)	39 (43.3)	0.512
No. (%) of patients with elevated C-reactive protein (>6 mg/dl)	40/93 (43)	40/73 (54.7)	0.132
No. (%) of patients with elevated procalcitonin (>0.1 ng/ml)	40/61 (65.5)	36/48 (75)	0.288
No. (%) of patients with abnormal IL-6^*h*^	62/78 (79.5)	61/70 (87.1)	0.215
No. (%) of patients with abnormal blood urea nitrogen^*i*^	43 (42.6)	44 (48.8)	0.375
No. (%) of patients with abnormal creatinine^*j*^	36 (35.6)	54 (60)	**0.001**
Median total leukocyte count (10^3^ cells/μl) (range)	10.7 (2.82–30.2)	10.96 (4–38.6)	0.707
Median platelet count (10^3^ cells/μl) (range)	188 (10–484)	158 (7–449)	**0.044**
Median C-reactive protein level (mg/dl) (range)	3 (0.021–25)	6.9 (0.085–37)	**0.025**
Median procalcitonin level (ng/ml) (range)	0.24 (0.01–100)	0.8 (0.01–100)	0.097
No. (%) of patients with disease severity			
Asymptomatic	9 (8.9)	3 (3.3)	0.338
Mild	32 (31.7)	25 (27.7)	
Moderate	20 (19.8)	19 (21.1)	
Severe/critical	40 (39.6)	43 (47.8)	
In-hospital complications and outcomes			
No. (%) of patients who required ventilatory support	47 (46.5)	52 (57.7)	0.121
No. (%) of patients who required ICU admission	69 (68.3)	70 (77.7)	0.143
Median duration of hospital stay (days) (range)	13 (1–35)	14 (1–46)	0.332
No. (%) of patients with ARDS	47 (46.5)	49 (54.4)	0.275
No. (%) of patients with shock	24 (23.7)	28 (31.1)	0.291
No. (%) of patients who died	33 (32.6)	36 (40)	0.307

a ALT, alanine aminotransferase; ARDS, acute respiratory distress syndrome; AST, aspartate aminotransferase; ICU, intensive care unit; IL-6, interleukin-6. Boldface type indicates statistical significance.

b Reference values are 12 to 15 g/dl for men and 13 to 17 g/dl for women.

c The reference range is 4 × 10^3^ to 11 × 10^3^ cells/μl.

d The reference range is 20 to 40%.

e The reference range is 150 × 10^3^ to 400 × 10^3^ cells/μl.

f The reference range is 5 to 40 U/liter.

g The reference range is 5 to 42 U/liter.

h The reference range is 5 to 15 pg/ml.

i The reference range is 10 to 50 mg/dl.

j The reference range is 0.5 to 1.2 mg/dl.

FTD assay results were not directly communicated to the treating clinicians; therefore, antibiotic optimization and modifications were not pursued. Seventy (77.7%) patients were admitted to the ICU, and 52 (57.8%) required ventilatory support. Of the 90 patients with coinfections, 36 (40%) died. We observed higher rates of ICU admissions in patients with coinfections than in those without coinfections, although the difference was not statistically significant (Table 5).

## DISCUSSION

In the present study, by rapid molecular testing, we identified coinfection with one or more pathogens in 46.5% (89/191) of patients with SARS-CoV-2. Additionally, five patients in our cohort had positive cultures for A. baumannii and K. pneumoniae, and two patients had active M. tuberculosis infection. In total, 47.1% (90/191) of patients were identified as having coinfections. This study’s results indicate a higher rate of coinfections between SARS-CoV-2 and other respiratory pathogens than those in a few previous reports (4, 7, 16–19). Zhang et al. reported bacterial coinfections in 7.7% of patients with SARS-CoV-2 (16). Additionally, a French study reported 28% coinfections at ICU admission of patients with COVID-19 (18). In contrast, in our cohort, the rate of coinfection was found to be higher, 47.1%. The higher rate of coinfection in this study could be due to the application of broad-range respiratory PCR that can detect a wide variety of pathogens, including viruses, bacteria, and fungi, compared to primary culture and PCR targeting a limited number of organisms. Meanwhile, in a Chinese study, a higher coinfection rate of 94.2% was reported (20). The variability in the overall proportions of coinfections in the present study could be attributed to the age group, comorbidities, and disease severity of patients; antibiotic exposure; the detection method employed; and spatiotemporal variations.

Staphylococcus aureus and Klebsiella spp. were the most commonly identified bacteria in patients with SARS-CoV-2, which agrees with previous reports (4, 5, 18, 21). These bacteria may significantly complicate infections in COVID-19 patients, especially in an ICU setting. Besides, infections in the lower respiratory tract caused by multidrug-resistant strains of these bacteria may cause substantial morbidity and mortality. Nevertheless, in the present study, we could not perform further molecular testing to identify the genes conferring drug resistance due to financial constraints. All four strains of A. baumannii isolated from patients were resistant to most antibiotics tested.

Moraxella catarrhalis in patients with COVID-19 has been reported in previous studies (21). The weakened immune response in COVID-19 patients, especially an inadequate CD8 T cell response, might have placed them at a high risk for this infection (21, 22). The prevalence of S. pneumoniae in the study population was 3.6%, mainly in middle-aged and older adults. Pneumococcal pneumonia has been reported in 1.2 to 3% of patients with SARS-CoV-2 in previous studies (23, 24). However, compared to the previous influenza pandemic, S. pneumoniae coinfection rates were low for COVID-19 (11, 20, 24). Evidence suggests that H. influenzae is one of the most common coinfecting bacterial pathogens in COVID-19 patients (18, 20, 21). In our study, nine patients were coinfected with H. influenzae, including two patients with H. influenzae type b (Hib). The detection of a microorganism from a respiratory specimen may not always be connected with an infection; nevertheless, it is difficult to differentiate between colonization and coinfection.

Legionella pneumophila was the only atypical bacterial pathogen identified in our study population. *Legionella* spp. can cause acute consolidating pneumonia in susceptible patients who have underlying health conditions or are immunodeficient (25, 26). Coinfection with M. pneumoniae or C. pneumoniae was not identified. However, we previously reported M. pneumoniae coinfection in a patient with SARS-CoV-2 using an in-house PCR (27).

In our cohort, 7.3% of patients were coinfected with other respiratory viruses. In contrast, a higher rate of coinfections of around 21% with viruses, including HRV, RSV, and non-SARS-CoV-2 *Coronaviridae*, was reported by Kim et al. (17). In previous reports, influenza A virus and RSV were commonly identified in patients with COVID-19 (5, 7, 28); however, HRV and HAdV were most frequently detected in the present study. We found two influenza B virus coinfection cases, which can increase the risk to COVID-19 patients. Coinfections with influenza virus and COVID-19 have been previously reported in the literature (29). In a Chinese study, 4.35% of patients with confirmed COVID-19 had coinfection with influenza virus (30). Therefore, physicians should suspect this clinical scenario as these viruses show similar transmission characteristics and common clinical features but differ considerably in their treatment. Coinfection with other respiratory viruses may lead to upper and lower respiratory tract infections and exhibit similar clinical presentations. Therefore, while diagnosing and treating SARS-CoV-2, other viral pathogens should be considered. Conversely, amid this pandemic, clinicians should also consider the possibility of COVID-19 regardless of positive results for other respiratory viruses (5, 20).

The implementation of an effective antibiotic stewardship program during the pandemic is of paramount importance (31). The inappropriate use of antibiotics for viral pneumonia may cause the emergence of antimicrobial resistance. The rates of detection of bacterial coinfections and the profile of bacteria identified in the present study encourage the systemic use of empirical antibiotic treatment in patients with severe COVID-19 pneumonia. Antibiotic de-escalation should be considered as soon as the respiratory PCR results are available.

### Clinical significance.

The process of concomitant infection by other respiratory pathogens and SARS-CoV-2 is still unclear. A few organisms identified in our study, including S. aureus, S. pneumoniae, H. influenzae, and M. catarrhalis, are commonly seen as colonizers in the upper respiratory tract and may increase the risk of invasive infections and serious complications. Oropharyngeal colonization by bacteria may appear to be a potential cause of ventilator-associated pneumonia (VAP), especially in ICU patients with SARS-CoV-2, causing increased hospital and ICU stays. Staphylococcus aureus, one of the most common coinfecting agents, has a reservoir in the oral cavity and is associated with oral disease conditions, including angular cheilitis, endodontic infections, parotitis, and osteomyelitis. Therefore, additional oral examinations may be recommended to identify this pathogen and prevent worsening the severity of COVID-19 (32).

Diffuse alveolar damage and bronchopneumonia caused by S. pneumoniae in a SARS-CoV-2 patient have been reported in the literature (24). Pneumococcal pneumonia may lead to bacteremia and secondary complications such as endocarditis, meningitis, and arthritis, especially in patients with certain medical conditions and risks for invasive pneumococcal infections, such as advanced or very young age, immunosuppression induced by HIV infection, renal and liver diseases, asplenia, and hematological malignancies. Invasive pneumococcal disease associated with COVID-19 has been previously reported (33). Haemophilus influenzae type b, one of the coinfecting pathogens in the present study, may also cause bacteremia and acute bacterial meningitis. This pathogen is associated with other conditions, including cellulitis, osteomyelitis, and epiglottitis. Moraxella catarrhalis can cause a variety of infections such as endocarditis, septicemia, and meningitis, especially in an immunocompromised individual.

Pneumocystis jirovecii colonization may occur in both immunocompromised and immunocompetent individuals. Coinfection with *P. jirovecii* and SARS-CoV-2 has been reported in a patient with progressive hypoxemic respiratory failure and CD4^+^ lymphocytopenia (34). Therefore, physicians may consider additional diagnostic testing such as serum β-d-glucan for *P. jirovecii*, especially when there are other characteristics supporting coinfections and classical risk factors for Pneumocystis pneumonia. Additionally, possible risks regarding health care transmission associated with bronchoscopy in these patients need to be considered (34).

Klebsiella pneumoniae was the second most common bacterium identified in this study. There is a risk of K. pneumoniae colonization and hospital-acquired infections (HAIs) in COVID-19 ICUs. Hand hygiene, patient isolation, and attempts to limit patient contact may reduce the risk of transmission of such HAI events (35). Community- and health care-associated infections due to hypervirulent strains of K. pneumoniae have been reported to cause disseminated and fatal infections involving the liver, lungs, central nervous system, and eyes. Therefore, K. pneumoniae infections, especially due to hypervirulent strains, may have the potential to complicate the course of COVID-19. Colonization by this pathogen is an established risk factor for invasive disease (36).

Acinetobacter baumannii contaminates hospital environments, can survive for a prolonged period on dry surfaces, and is responsible for nosocomial infections, including hospital-acquired pneumonia, bloodstream infections, urinary tract infections, and wound infections. In this study, four patients had multidrug-resistant A. baumannii infection, which might have been acquired nosocomially. Risk factors for the acquisition of A. baumannii include cardiovascular system disease, endotracheal intubation, immunosuppression, and prior use of antibiotics. To interrupt the transmission of this pathogen in hospitals, strict adherence to infection control practices is essential (37). Bacterial, viral, and fungal infections, including infections in regions of endemicity, should be considered while managing patients with COVID-19, and the presence of these organisms in SARS-CoV-2-infected individuals requires proper evaluation and treatment in a timely fashion.

### Limitations.

Our study has a few limitations. First, the analysis was limited to detect selected coinfection patterns included in the multiplex respiratory PCR panel. Second, respiratory PCR was performed on oropharyngeal/nasal swabs in this study, which might have impacted the prevalence of the organisms detected and encountered in the patient population. The collection of invasive respiratory samples in COVID patients was restricted to prevent aerosol-generating procedures that pose a significant risk to health care staff and patients. Third, the respiratory PCR results were not communicated to treating clinicians to optimize antimicrobial treatment. Fourth, it is difficult to differentiate whether the bacterial infections reported in this study are of a community-acquired or nosocomial origin. The patient might have harbored the organism before the viral infection, or the pathogen might be part of an underlying chronic illness or might be picked up nosocomially. Finally, the shedding of respiratory pathogens does not always represent the shedding of viable or infectious viruses and might represent low-level residual nonviable viruses. Hence, physicians should consider the clinical significance of these pathogens when treating critically ill patients with SARS-CoV-2 infection.

In summary, using rapid molecular screening, we identified bacterial-viral coinfections in a high proportion of patients with SARS-CoV-2 infection. Due to intersecting signs and symptoms of fever, chills, respiratory distress, and throat pain, it is challenging to differentiate among flu, other respiratory illnesses, and COVID-19. Syndromic testing for multiple respiratory pathogens in hospitalized patients with SARS-CoV-2 infection allows the rapid detection of other pathogens and select interventions, including pathogen-targeted therapy or isolation. It may also be helpful from a prognostic standpoint. Application of respiratory PCR and initiation of narrow-spectrum agents are the mainstays of antibiotic stewardship in patients with severe COVID-19. Further large-scale studies are needed to determine the actual prevalence of coinfections, predictors, and significance of these infections in critically ill patients’ prognoses.

### Conclusions.

In this observational study, we report bacterial-viral coinfections in 47.1% of patients with SARS-CoV-2 infection. The bacterial coinfections were mostly related to K. pneumoniae, S. aureus, H. influenzae, and S. pneumoniae. Legionella pneumophila was the only atypical bacterium identified in our patient population. The common concomitant viral pathogens in our cohort were HRV, HAdV, and non-SARS-CoV-2 *Coronaviridae*. Clinicians should anticipate and must have a high index of suspicion for coinfections and secondary infections in patients with SARS-CoV-2 pneumonia. Screening for other respiratory pathogens during the clinical course of critically ill COVID-19 patients is critical for appropriate diagnosis and treatment. Empirical antibiotic treatment, if indicated, should be prescribed to critically ill patients with SARS-CoV-2 infection, with rapid de-escalation based on respiratory PCR/culture results.

## MATERIALS AND METHODS

### Study population.

The study was conducted at the All-India Institute of Medical Sciences (AIIMS), a large tertiary care referral hospital located in New Delhi, India, which has dedicated COVID care units. From 1 June 2020 through 31 January 2021, 191 patients (median age, 50 years; range, 15 to 86 years) admitted with COVID-19 were enrolled in this study. The Institute Ethical Committee of the AIIMS approved the study protocol (reference no. IEC-287/17.04.2020, RP-35/2020, and OP-07-05.02.2021). Laboratory confirmation of COVID-19 was achieved by either real-time reverse transcription-PCR (RT-PCR), a cartridge-based nucleic acid amplification test (CB-NAAT), or a rapid antigen test on combined oropharyngeal/nasal swab specimens. Patient demographic and clinical details; comorbid conditions; laboratory results, including microbiological analysis; in-hospital management; and outcomes were collected using a standard questionnaire.

### Sample collection and molecular testing.

Combined oropharyngeal/nasal swabs were collected from patients and transported to the laboratory, where an FTD respiratory pathogen 33 (FTD-33) assay (Fast Track Diagnostics, Luxembourg) was performed. For most patients (146 [76.4%]), the assay was performed after ≥48 h of hospital admission. Total nucleic acid was extracted from the oropharyngeal/nasal swabs using a QIAamp MinElute virus spin kit (Qiagen, Hilden, Germany). The FTD assay is a one-step RT-PCR containing primer-probe mixtures for the simultaneous amplification of 33 respiratory pathogens: influenza A virus (IAV); influenza A (H1N1) virus (swine lineage) [IAV(H1N1) swl]; influenza B virus (IBV); influenza C virus (IVC); human coronaviruses (HCoVs) NL63, 229E, OC43, and HKU1; human parainfluenza viruses (HPIVs) 1, 2, 3, and 4; human metapneumoviruses (HMPVs) A and B; human rhinovirus (HRV); human respiratory syncytial viruses (HRSVs) A and B; human adenovirus (HAdV); enterovirus (EV); human parechovirus (HPeV); human bocavirus (HBoV); *P. jirovecii*; M. pneumoniae; C. pneumoniae; S. pneumoniae; Haemophilus influenzae type b; S. aureus; Moraxella catarrhalis; *Bordetella* spp.; K. pneumoniae; L. pneumophila-L. longbeachae; Salmonella spp.; Haemophilus influenzae (non-type b); and equine arteritis virus (EAV), which served as an internal control (IC).

The FTD-33 assay was performed according to the manufacturer’s instructions. For PCR, 10 μl of extracted nucleic acid samples was mixed with 20 μl of master mix containing 12.5 μl of buffer, 1.5 μl of the primer-probe mix, and 1 μl of the enzyme. The multiplex real-time RT-PCR thermal profile was as follows: 50°C for 15 min, 94°C for 1 min, 40 cycles of 94°C for 8 s, and 60°C for 1 min. A sample was considered positive for a pathogen for any sigmoidal curve within a cycle threshold (*C_T_*) value of <40. An IC assessed both nucleic acid extraction and PCR inhibition. The IC was extracted with the specimens and used with each PCR run along with positive and negative controls provided by the manufacturers.

### Statistical analysis.

All continuous variables are expressed as medians (interquartile ranges [IQRs]), and categorical variables are expressed as numbers (percentages). The chi-square test or Fisher’s exact test was used to assess the association between the two categorical variables. A *t* test or Wilcoxon rank-sum test was used to compare the continuous variables between two independent groups according to the data distribution, and for more than two groups, analysis of variance (ANOVA) was used. The correlation between two continuous variables was determined by Pearson’s correlation or Spearman rank correlation as appropriate. Statistical significance was considered at a *P* value of <0.05. All statistical analysis was performed using STATA statistical software (14.2).
